# Supplementary material for: Assessing generalizability of an AI-based visual test for cervical cancer screening
Source: PLOS Digit Health. 2024 Oct 2;3(10):e0000364. doi: 10.1371/journal.pdig.0000364 (PMC11446437; doi:10.1371/journal.pdig.0000364)
Supplement: S1 Text — (DOCX) [file pdig.0000364.s001.docx]

**S1 TEXT: SUPPLEMENTARY METHODS AND RESULTS – REPEATABILITY AND CLASSIFICATION PERFORMANCE ANALYSIS**

In addition to the primary analysis conducted in the manuscript, we further evaluated the improvements in repeatability and classification performance imparted by the two key innovations of our model, multiclass classification (vs. binary) and the incorporation of Monte-Carlo (MC) dropout. This was assessed by four distinct model runs which only differed in terms of these two design choices, with all other hyperparameters kept constant: (i) binary; (ii) binary with MC dropout; (iii) multiclass; and (iv) multiclass with MC dropout. Each of these models were trained on a dataset that comprised of the “SEED” dataset together with a small number of “EXT” (J8) images added in 2n N : 2n I : 1n P ratio of ground truths at the women level, for a total of n = 26 women, and tested on a held-aside “EXT” (J8) dataset. For each of these cases, we first assessed the repeatability of these models via Bland-Altman plots and the corresponding 95% LoA (S1 Fig a), test-retest score plots (S1 Fig b), and the degree of extreme disagreement (% 2-class disagreement between image pairs across women) (S1 Fig c 1) We assessed the classification performance of each of these models via the total degree of extreme misclassifications, the % precancer+ misclassified as normal, and the % normal misclassified as precancer+ (S1 Fig c 2-4).

We hypothesized that the two key design innovations utilized in our model, namely multiclass classification (vs. binary) and the incorporation of MC dropout are optimized for both improved repeatability of predictions across multiple images from the same woman, as well as improved class discrimination, which subsequently carries over well to “external” data (“EXT”) in the form of a new device (J8). This is evidenced in S1 Fig, which highlights key classification and repeatability metrics for each of the four models under investigation namely (i) binary, (ii) binary with MC dropout, (iii) multiclass and (iv) multiclass with MC dropout (our model). Panel (a) highlights the improvement in repeatability via decrease in the 95% LoA on the Bland Altman plot; specifically, the corresponding 95% LoA values are (i) 0.75, (ii) 0.50, (iii) 0.51, and (iv) 0.41 respectively. Both the binary to multiclass transition and the no dropout to MC dropout transition improve repeatability, with the multiclass MC model achieving the best repeatability among all models. This is reinforced by the test-retest score plot in (b), which highlights progressively stronger alignment to the diagonal representing no difference between image 1 and image 2 score at the patient level, from (i) through to (iv). Panel (c) (1) also highlights that the multiclass MC model (our model) achieves the smallest degree of extreme disagreement on repeat images per woman (0.25%), i.e., the fewest women for whom image 1 is predicted “normal” and image 2 is predicted “precancer+” and vice versa. Taken together, panel (a), panel (b) and panel (c) (1) suggest that our multiclass model with MC dropout is strongly optimized for improved repeatability.

Panel (c) (2) – (4) highlight the improvement in classification performance, represented by successive decrease in % extreme misclassification, % normal misclassified as precancer+, and % precancer+ misclassified as normal, as we go from binary to binary MC to multiclass to a multiclass MC model. The incorporation of multi-level ground truth delineations during our model selection approach was designed to (1) account for the inherent clinician uncertainty or the equivocal nature of certain pathologies (e.g., ASCUS in the Bethesda system) and (2) ensure reduction of grave errors or extreme misclassifications – Panel (c) (2) – (4) highlight that this is strongly achieved, with the multiclass MC model achieving the lowest % ext. mis. = 3.96%, % p as n = 8.29%, and % n as p = 1.30% respectively (purple bars in 1 – 4).

Overall, we highlight that our model achieves strong repeatability of predictions when evaluated on external data. In particular, our model makes reliable, consistent predictions on external data irrespective of the axis of data heterogeneity i.e., on individuals from new geographies or new devices. This is achieved even in the absence of retraining, and remains relatively constant throughout incremental retraining, as Table 2 highlights. This is largely attributable to the presence of MC dropout and the dedicated optimization of repeatability as a selection criterion during model optimization.
