# Supplementary figures and images for: Assessing generalizability of an AI-based visual test for cervical cancer screening
Source: PLOS Digit Health. 2024 Oct 2;3(10):e0000364. doi: 10.1371/journal.pdig.0000364 (PMC11446437; doi:10.1371/journal.pdig.0000364)

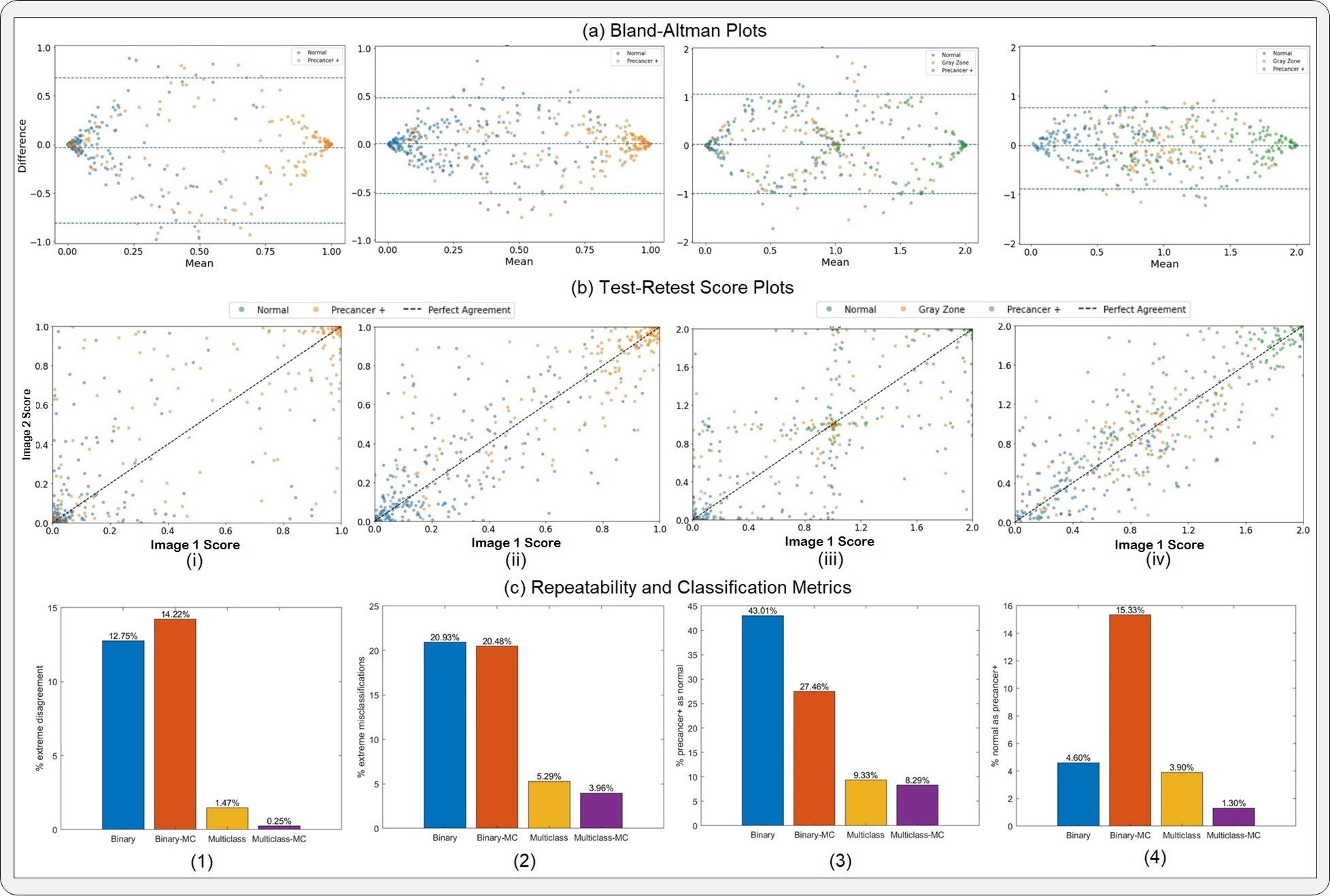

Supplement: S1 Fig — (a) Bland-Altman plots; and (b) Test-Retest score plots for each of the four models under investigation namely (i) binary; (ii) binary with MC dropout; (iii) multiclass; and (iv) multiclass with MC dropout (our model), in order to assess the relative impact of the key design choices of our model. Panel (c) (1) highlights the % extreme disagreement (proportion of women for whom the model predicts “normal” for image 1 and “precancer+” for image 2 and vice-versa) for the each of the four models (repeatability), while Panel (c) (2)–(4) highlights relevant classification metrics, including (2) the % extreme misclassification (precancer+ misclassified as normal and vice-versa); (3) the % precancer+ misclassified as normal; and (4) the % normal misclassified as precancer+, for each of the four models. “Gray Zone” = “Indeterminate”. (TIF) [file pdig.0000364.s002.tif]

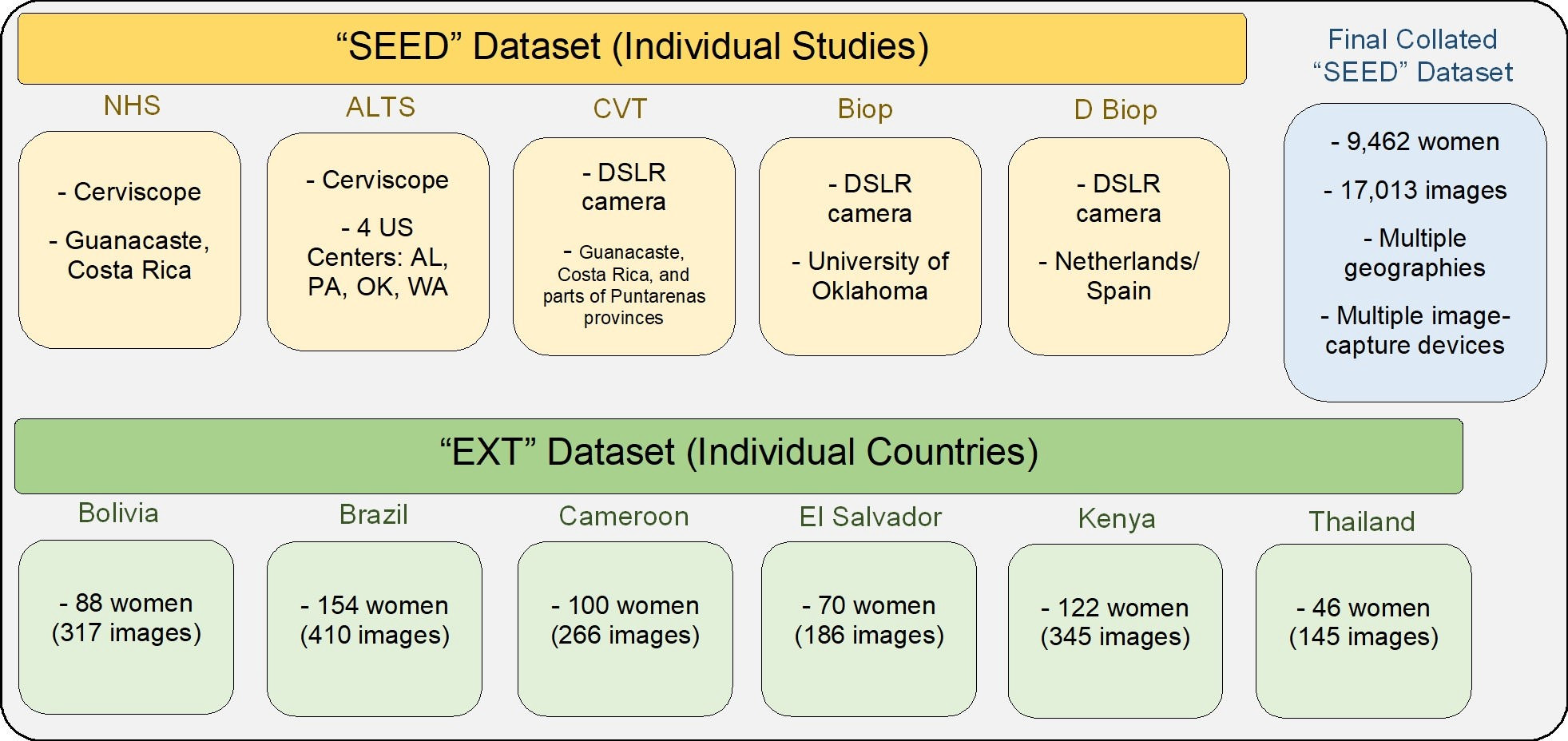

Supplement: S2 Fig — The top panel highlights the five different studies (NHS, ALTS, CVT, Biop and D Biop) used to generate the final collated “SEED” dataset (top right) on which our model was trained and internally validated. The bottom panel highlights the six different countries / geographies included in the “EXT” dataset, all comprising of images acquired using a Samsung Galaxy J8 smartphone, on which our model was externally validated. (TIF) [file pdig.0000364.s003.tif]
