# Supplementary material for: Assessing generalizability of an AI-based visual test for cervical cancer screening
Source: PLOS Digit Health. 2024 Oct 2;3(10):e0000364. doi: 10.1371/journal.pdig.0000364 (PMC11446437; doi:10.1371/journal.pdig.0000364)
Supplement: S1 Table — Our model performs consistently well on “internal” data, even when retrained with added “external” images from the “EXT” dataset. Metrics are reported for each of the model runs involving incremental additions of images from the “EXT” (J8) dataset at the woman level, in a 2n normal (N): 2n indeterminate (I): 1n precancer+ (P) ratio of ground truth class, where n = # of precancer+ women added, as shown on the leftmost column. % values are rounded to 1 decimal place, while numeric values are rounded to 2 decimal places. (DOCX) [file pdig.0000364.s004.docx]

| S1 Table: Classification and Repeatability Metrics on “SEED” Test Set | | | | | | |
| --- | --- | --- | --- | --- | --- | --- |
| Model # | **Classification** | | | | **Repeatability** | |
|  | AUROC  normal vs. rest | AUROC  precancer+ vs. rest | % ext. mis. | % tot. mis. | % ext. dis. | 95% LoA |
| Add 05 | 0.88 | 0.86 | 3.6% | 28.1% | 0.4% | 0.24 |
| Add 13 | 0.88 | 0.84 | 3.8% | 29.0% | 0.9% | 0.25 |
| Add 16 | 0.88 | 0.87 | 5.9% | 30.9% | 0.9% | 0.28 |
| Add 18 | 0.88 | 0.87 | 2.9% | 27.0% | 1.3% | 0.25 |
| Add 21 | 0.88 | 0.87 | 4.0% | 30.6% | 0.5% | 0.27 |
| Add 23 | 0.88 | 0.86 | 6.5% | 32.5% | 0.7% | 0.30 |
| Add 26 | 0.88 | 0.87 | 3.4% | 30.8% | 1.0% | 0.24 |
| Add 28 | 0.88 | 0.86 | 5.6% | 31.1% | 0.5% | 0.25 |
| Add 41 | 0.88 | 0.87 | 5.6% | 32.9% | 0.9% | 0.25 |
| Add 45 | 0.88 | 0.86 | 3.4% | 29.8% | 0.8% | 0.24 |
| Add 50 | 0.88 | 0.86 | 2.7% | 28.4% | 0.4% | 0.22 |
| Add 55 | 0.88 | 0.85 | 6.2% | 33.4% | 0.6% | 0.28 |
| Add 60 | 0.88 | 0.87 | 3.2% | 28.3% | 1.0% | 0.23 |
| Add 65 | 0.88 | 0.86 | 5.2% | 29.1% | 0.5% | 0.22 |
| Add 70 | 0.88 | 0.87 | 3.6% | 29.8% | 0.8% | 0.27 |
